# Evaluation of drug-resistant tuberculosis treatment outcome in Portugal, 2000–2016

**DOI:** 10.1371/journal.pone.0250028

**Published:** 2021-04-20

**Authors:** Olena Oliveira, Rita Gaio, Margarida Correia-Neves, Teresa Rito, Raquel Duarte

**Affiliations:** 1 Life and Health Sciences Research Institute (ICVS), School of Medicine, University of Minho, Braga, Portugal; 2 ICVS/3B, PT Government Associate Laboratory, University of Minho, Braga/Guimarães, Portugal; 3 EPIUnit, Instituto de Saúde Pública da Universidade do Porto, Porto, Portugal; 4 Department of Mathematics, Faculty of Sciences, University of Porto, Porto, Portugal; 5 Centre of Mathematics, University of Porto, Porto, Portugal; 6 Centre of Molecular and Environmental Biology (CBMA), University of Minho, Braga, Portugal; 7 Clinical Epidemiology, Predictive Medicine and Public Health Department, Faculty of Medicine, University of Porto, Porto, Portugal; 8 Pulmonology Unit, Centro Hospitalar de Vila Nova de Gaia/Espinho EPE, Vila Nova de Gaia, Portugal; All India Institute of Medical Science - Bhopal, INDIA

## Abstract

Treatment of drug-resistant tuberculosis (TB), which is usually less successful than that of drug-susceptible TB, represents a challenge for TB control and elimination. We aimed to evaluate treatment outcomes and to identify the factors associated with death among patients with MDR and XDR-TB in Portugal. We assessed MDR-TB cases reported for the period 2000–2016, using the national TB Surveillance System. Treatment outcomes were defined according to WHO recommendations. We identified the factors associated with death using logistic regression. We evaluated treatment outcomes of 294 MDR- and 142 XDR-TB patients. The treatment success rate was 73.8% among MDR- and 62.7% among XDR-TB patients (p = 0.023). The case-fatality rate was 18.4% among MDR- and 23.9% among XDR-TB patients. HIV infection (OR 4.55; 95% CI 2.31–8.99; *p* < 0.001) and resistance to one or more second-line injectable drugs (OR 2.73; 95% CI 1.26–5.92; *p* = 0.011) were independently associated with death among MDR-TB patients. HIV infection, injectable drug use, past imprisonment, comorbidities, and alcohol abuse are conditions that were associated with death early on and during treatment. Early diagnosis of MDR-TB and further monitoring of these patients are necessary to improve treatment outcome.

## Introduction

Tuberculosis (TB) treatment success (the percentage of cured patients and those with treatment completed) is one indicator for monitoring implementation of the End TB Strategy. Globally, the recommended target level for 2025 is above 90% [1]. In the European Union, the treatment success rate for the 45,499 TB cases treated in 2017 was 67.6%, still standing far from the established goal. Moreover, the latest surveillance data (2018) for patients with drug-resistant TB shows lower treatment success rates: 48.1% for multidrug-resistant TB (MDR-TB), defined by resistance of *Mycobacterium tuberculosis* (Mtb) to isoniazid and rifampicin, and 37.4% for extensively drug-resistant TB (XDR-TB), defined as MDR-TB plus resistance to at least one of the fluoroquinolones and one of the injectable drugs [2]. Death is a more frequent unfavorable outcome among those with MDR- or XDR-TB than with susceptible TB (17.1%, 21.8%, and 6.9%, respectively) [2].

The treatment of MDR/XDR-TB requires the use of bactericidal and bacteriostatic drugs for long periods. Treatment success depends not only on the choice of an effective treatment regimen but also on the patient monitorisation and the management of therapeutics adverse events and comorbidities, potential drug-drug interactions, and even the patient’s tolerability to the drug regimen implemented [3].

In Portugal, TB cases are managed mainly in TB Outpatient Centres. As a specific strategy to MDR-TB control, in 2007, the National Reference Centre was created to monitor and support the treatment of MDR/XDR-TB cases, producing the national guidelines and recommendations. Later, to support the implementation of these standard procedures, to decentralize this approach and to facilitate accessibility to all, the Regional Reference Centres were created. Although these Centres currently operate in each of the seven health regions of the country, they started working at different times. While the first Centre opened in the Northern Region in 2009 [4,5], the Centre in Lisbon and Tagus Valley Region, with the highest TB burden in the country, started to work only in 2013 [6]. In Portugal, all TB patients receive free treatment from the National Health System under the National Tuberculosis Program. Hospitalization is the first choice at the start of MDR-TB treatment, and the patient remains hospitalized until smear sputum conversion. The Regional Reference Centres are responsible for the clinical management of patients during the entire treatment course, including the choice of the treatment regimen. Adequate regimens are based on current MDR-TB treatment guidelines [7,8] and adjusted according to the clinical and microbiological response along with drug susceptibility testing (DST) results. Directly Observed Therapy (DOT) is provided throughout the treatment at the primary care level [4,5].

Following our earlier analysis evaluating treatment outcomes of MDR-TB patients in Portugal [9], here we updated information to give a complete assessment of the disease along 17 years of cases reported in Portugal. Furthermore, we assessed case-fatality rate and identified the factors associated with death in MDR- and XDR-TB patient groups.

## Methods

### Data collection

We selected MDR-TB cases diagnosed in Portugal from January 2000 until December 2016 from the national TB Surveillance System (SVIG-TB). We evaluated patients with known treatment outcomes and second-line drug resistance profiles, dividing those into groups: MDR- and XDR-TB cases.

Information collected included demographic and clinical characteristics of each case: age, sex, country of origin, addictions (e.g., drug or alcohol abuse), HIV status, living conditions (e.g., being a prisoner, living in a community residence, homelessness), comorbidities (diabetes, silicosis, chronic obstructive pulmonary disease, liver disease or neoplasia), previous TB treatment and site of infection. We also collected dates of the onset of symptoms, diagnosis of TB, and treatment initiation.

### Diagnosis and treatment

MDR-TB diagnosis requires a positive culture or detection of both acid-fast bacilli by microscopy and an Mtb-specific nucleic acid amplification testing, followed by detection of resistance to isoniazid and rifampicin by genotypic and phenotypic methods [2]. We included in our study culture-positive MDR-TB cases tested for resistance to first- and second-line anti-TB drugs by phenotypic methods, the conventional gold standard. All tests were performed in laboratories integrated into the national network, periodically certified and checked. Mtb strains that revealed resistance to isoniazid and rifampicin were tested for second-line anti-TB drugs in the TB National Reference Laboratory (Instituto Nacional de Saúde Ricardo Jorge: INSA) in Porto, which is also a World Health Organization (WHO) Supranational Reference Laboratory.

HIV testing is done routinely, using an opt-out strategy (patients were informed that an HIV test will be conducted and that they may decline or defer) [10]. The treatment regimen is designed according to WHO guidelines. Treatment begins with a standard or empirical regimen until DST for second-line drugs results are available. Afterwards, individually tailored regimens that take into account the drug resistance patterns are used. Treatment continues for at least 18 months after culture conversion [11–13]. The patients are hospitalized until smear conversion and then followed in one of the Regional Reference Centre. Directly observed treatment is performed during the entire treatment.

### Treatment outcomes

The treatment outcomes were defined according to WHO recommendations as *cured*, *treatment completed*, *treatment failed*, *death*, and *lost to follow-up* [7,14], and reported to the National TB Surveillance System. The sum of *cured* (treatment completed and three or more consecutive cultures taken at least 30 days apart are negative after the intensive phase) and *treatment completed* (treatment completed but no record that three or more consecutive cultures taken at least 30 days apart are negative after the intensive phase) is considered as treatment success. That is, treatment success includes *treatment completed* with or without three or more consecutive cultures taken at least 30 days apart, which are negative after the intensive phase. Although in our study no cured was registered during the studied period, it will not affect estimated success rate. *Treatment failure*, *death* and *lost to follow-up* were considered as unfavorable treatment outcomes.

The percentage of cases for each outcome was determined considering the total number of patients who started treatment over studied period.

### Statistical analysis

We describe patient’s characteristics through absolute and relative frequencies for categorical variables as gender, country of origin, HIV status, alcohol abuse, injectable drug use, prisoner, community residence, homelessness, comorbidity, chest radiography, previous TB treatment, site of disease. Median with interquartile range (IQR) was used for continuous variables as age (years), delay in diagnosis and treatment (days) and duration of treatment (months). Delay in diagnosis and treatment was defined as the period from the date of the onset of symptoms until the diagnosis of TB and treatment initiation. We compared the prevalence of these characteristics between patients grouped according to second-line drug resistance profile and between MDR-TB patients that died within and after the first six months of treatment, using the Chi-squared test (or Fisher’s test, if appropriate) for categorical variables. The Mann–Whitney U-test (or median test) was used to compare continuous variables. We estimated treatment success rate by year and we compared their medians before and after 2008, year that corresponds to half of the study period and when some measures of MDR-TB control were taken in Portugal. We also estimated treatment success rate over the study period and we compared it among patient groups. We used the Chi-squared test (or Fisher’s test, if appropriate) to compare patient groups’ treatment outcomes. Univariate and multivariate logistic regression was conducted to identify death factors during treatment among patients in each drug resistance categories. Crude and adjusted odds ratios (ORs) and 95% confidence intervals (CIs) were determined. Statistical analyses were performed with SPSS version 18.0 (PASW Statistics 18), and p-values below 0.05 were considered statistically significant.

### Ethical considerations

Ethical approval and informed consent were not required, as the patient data, collected by an official national surveillance system, were anonymized following the ethical research guidelines in Portugal.

## Results

### Dataset characterization

**Fig 1:**
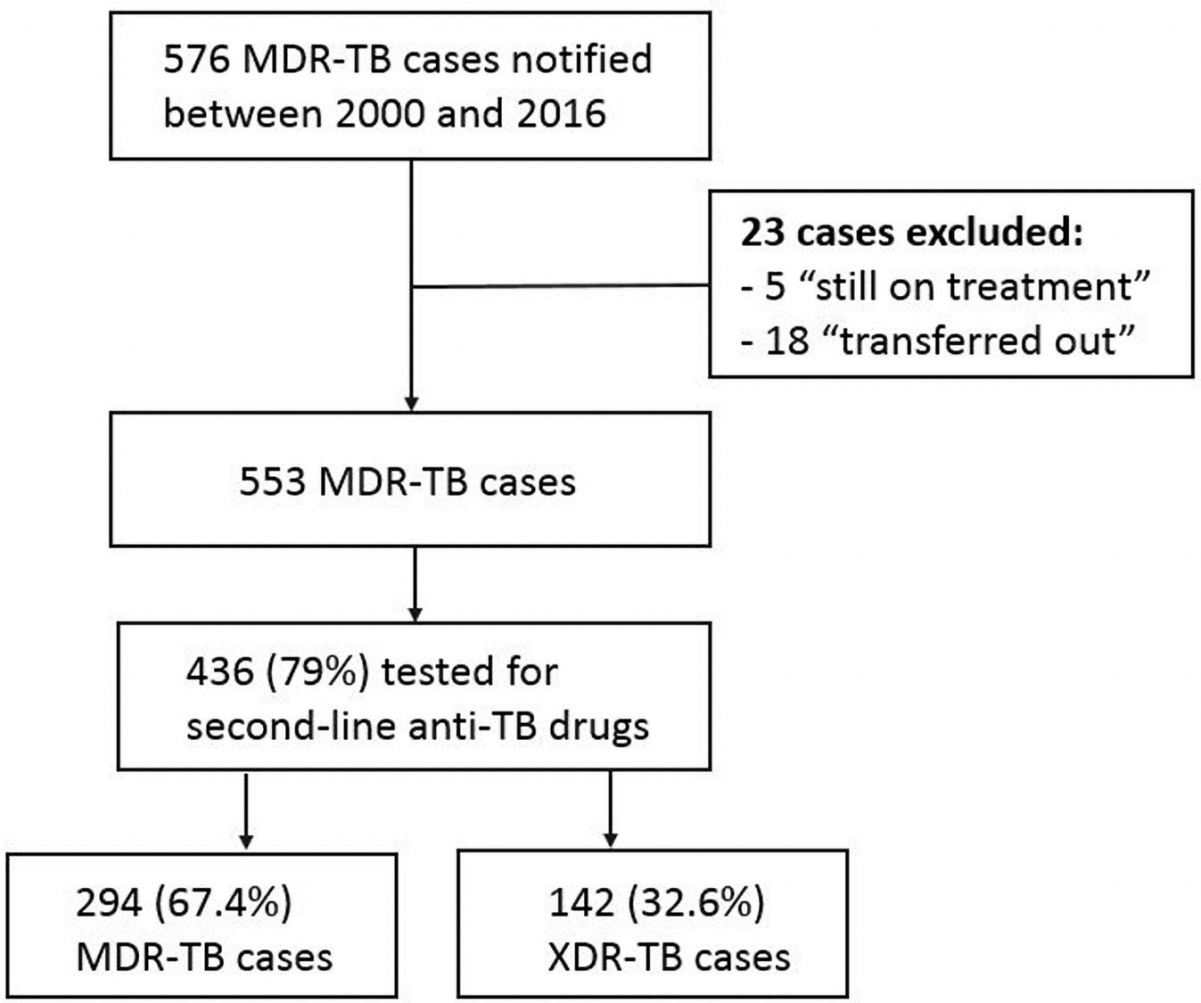
Flowchart of the cases included in the analysis, considering MDR-TB cases reported between 2000 and 2016 in Portugal. MDR-TB = multidrug-resistant tuberculosis; XDR-TB = extensively drug-resistant tuberculosis.

In Portugal, from January 2000 until December 2016, 576 MDR-TB cases were diagnosed. We evaluated 436 cases, excluding patients with unknown treatment outcomes and second-line drug resistance profiles. Of them, 294 (67.4%) cases were MDR-TB, and 142 (32.6%) cases were XDR-TB (Fig 1).

Demographic and clinical characteristics of the patients are shown in Table 1. Compared to MDR-TB, XDR-TB patients presented higher prevalence of alcohol abuse (34.1%), injectable drug use (30.8%), past or present imprisonment (12.7%) and previous TB treatment (54.9%) (Table 1).

**Table 1:** Characteristics of multidrug-resistant and extensively drug-resistant tuberculosis patients, considering the cases reported in Portugal between 2000 and 2016 (n = 436).

Patient’s characteristics		Total N	MDR-TB		XDR-TB		p-value
			n or M	% or IQR	n or M	% or M	
**Categorical variables**							
Gender	Female	436	93	31.6	37	26.1	0.280
	Male		201	68.4	105	73.9	
Country of origin	Native	435^a^	213	72.7	114	80.3	0.110
	Foreign-born		80	27.3	28	19.7	
HIV status	Negative	436	224	76.2	98	69.0	0.138
	Positive		70.0	23.8	44	31.0	
Alcohol abuse^b^	No	387^a^	203	77.8	83	65.9	**0.018**
	Yes		58	22.2	43	34.1	
Injectable drug use^b^	No	392^a^	224	85.5	90	69.2	**0.001**
	Yes		38	14.5	40	30.8	
Imprisonment	No	383^a^	247	96.1	110	87.3	**0.003**
	Yes		10	3.9	16	12.7	
Community residence^b^	No	380^a^	244	95.3	118	95.2	1.000
	Yes		12	4.7	6	4.8	
Homelessness	No	380^a^	248	96.9	120	96.8	1.000
	Yes		8	3.1	4	3.2	
Comorbidities	No	436	252	85.7	121	85.2	1.000
	Yes		42	14.3	21	14.8	
Chest radiography	No cavitation	401^a^	123	44.7	58	46.0	0.892
	Cavitation		152	55.3	68	54.0	
Previous TB treatment	No	436	187	63.6	64	45.1	**<0.001**
	Yes		107	36.4	78	54.9	
Site of disease	Pulmonary	435^a^	278	94.6	131	92.9	0.643
	Extra-pulmonary		16	5.4	10	7.1	
**Continuous variables**							
Age (years)	Median, IQR	436	41.0	20.0	40.0	16.0	0.611
Delay in TB diagnosis and treatment (days)	Median, IQR	333^a^	73.0	84.0	71.0	63.0	0.941
Treatment duration (months)	Median, IQR	436	20.4	13.2	23.4	10.7	**<0.001**

n = number of cases; M = median; IQR = interquartile range; HIV = human immunodeficiency virus; TB = tuberculosis; MDR-TB = multidrug-resistant tuberculosis; XDR-TB = and extensively drug-resistant tuberculosis.

^a^ Data missing for: Country of origin (n = 1; 0.2%), alcohol abuse (n = 49; 11.2%), injectable drug use (n = 44; 10.1%), prisoners (n = 53; 12.2%), community residence (n = 56; 12.8%), homelessness (n = 56; 12.8%), chest radiography (n = 35; 8.0%), site of disease (n = 1; 0.2%), delay in TB diagnosis and treatment (n = 103; 23.6%).

^b^ Self-reported.

### Treatment and treatment outcomes

In our study, median TB diagnostic delay, which was calculated for patients with available date for the onset of symptoms (n = 333; 76.4%), was 72 days. There were no significant differences in TB diagnostic delay between MDR- and XDR-TB (73 days and 71 days; *p* = 0.941; Table 1).

All 436 patients started treatment on the day of diagnosis of TB. Initial treatment regimen data was available for 414 (95.0%) patients. Of them, 170 (41.1%) received second-line anti-TB drugs in the initial treatment regimen.

Duration of treatment among MDR-TB patients was 20.4 months and 23.4 months among XDR-TB patients (*p* < 0.001) (Table 1).

The treatment success rate tended to increase since 2000 to 2016 (S1 Fig). We compared the medians of treatment success rate before and after 2008 and we found no statistically significant difference between them (67.5% and 78.6% respectively; *p* = 0.153).

The treatment success rate over the study period was superior among MDR than XDR-TB patients (73.8% and 62.7%; *p* = 0.023; Table 2). Among unfavorable treatment outcomes, *death* was more frequent than *treatment failure* or *loss to follow-up* in both groups. The case-fatality rate among MDR- and XDR-TB patients was 18.4% and 23.9% respectively (*p* = 0.218; Table 2).

**Table 2:** Treatment outcomes among multidrug-resistant and extensively drug-resistant tuberculosis patients who started treatment between 2000 and 2016 (n = 436).

Treatment outcomes		Total		MDR-TB		XDR-TB		*p-*value
		n	%	n	%	n	%	
**Treatment success**	Treatment completed	306	70.2	217	73.8	89	62.7	**0.023**
**Unfavorable outcomes**	Treatment failed	16	3.7	8	2.7	8	5.6	0.213
	Lost to follow-up	26	6.0	15	5.1	11	7.7	0.381
	Death	88	20.2	54	18.4	34	23.9	0.218

n = number of cases; MDR-TB = multidrug-resistant tuberculosis; XDR-TB = and extensively drug-resistant tuberculosis.

Additionally, we evaluated treatment outcomes among MDR-TB patients, dividing cases into groups: MDR-TB without additional second-line drug resistance, MDR-TB with additional resistance to one or more second-line injectable drugs (pre-XDR_SLID_-TB) and MDR-TB with additional resistance to one or more fluoroquinolones (pre-XDR_FQ_-TB) (S1 Table).

We found that pre-XDR_SLID_-TB patients had worse treatment outcome than patients from other drug-resistance groups: the treatment success rate was 55.6% (*p* = 0.002) and the case-fatality rate was 31.0% (*p* = 0.065) among them (S1 Table). However, due to the small numbers of patients in with pre-XDR_SLID_- and pre-XDR_FQ_-TB, we decided not to go ahead with looking for factors associated with death in these groups, but to include these resistance profiles as independent variables in the analysis of factors associated with patient death among MDR- TB.

### Factors associated with patient death

We assessed separately factors associated with patient death among MDR- and XDR-TB patients (Tables 3 and 4).

**Table 3:** Factors associated with death among patients with multidrug-resistant tuberculosis (n = 294).

Factors	Death		Univariate analysis		Multivariate analysis	
	No n (%)	Yes n (%)	OR (95% CI)	*p*-value	OR (95% CI)	*p*-value
Age (years), median (IQR)	41.0(18)	40.5(29)	1.02(0.99–1.04)	0.111		
Gender						
Female	80(86.0)	13(14.0)	Ref			
Male	160(79.6)	41(20.4)	1.58(0.80–3.11)	0.189		
Country of origin						
Native	174(81.7)	39(18.3)	Ref			
Foreign-born	65(81.2)	15(18.8)	1.03(0.53–1.99)	0.931		
HIV status						
Negative	196(87.5)	28(12.5)	Ref		Ref	
Positive	44(62.9)	26(37.1)	4.14(2.21–7.74)	**<0.001**	4.55(2.31–8.99)	**<0.001**
Alcohol abused						
No	166(81.8)	37(18.2)	Ref			
Yes	48(82.8)	10(17.2)	0.94(0.43–2.02)	0.963		
Injectable drug used						
No	196(87.5)	28(12.5)	Ref			
Yes	20(52.6)	18(47.4)	6.30(2.98–13.36)	**<0.001**		
Imprisonment						
No	208(84.2)	39(15.8)	Ref			
Yes	6(60.0)	4(40.0)	3.56(0.96–13.19)	0.058		
Community residence						
No	204(83.6)	40(16.4)	Ref			
Yes	10(83.3)	2(16.7)	1.02(0.22–4.83)	0.980		
Homelessness						
No	207(83.5)	41(16.5)	Ref			
Yes	7(87.5)	1(12.5)	0.72(0.09–6.02)	0.763		
Comorbidities						
No	211(83.7)	41(16.3)	Ref			
Yes	29(69.0)	13(31.0)	2.31(1.11–4.81)	**0.026**		
Chest radiography						
No cavitation	98(79.7)	25(20.3)	Ref			
Cavitation	131(86.2)	21(13.8)	0.63(0.33–0.19)	0.153		
Previous TB treatment						
No	156(83.4)	31(16.6)	Ref			
Yes	84(78.5)	23(21.5)	1.38(0.76–2.51)	0.296		
Site of disease						
Pulmonary	228(82.0)	50(18.0)	Ref			
Extra-pulmonary	12(75.0)	4(25.0)	1.52(0.47–4.91)	0.484		
Pre-XDR_SLID_-TB						
No	190(85.6)	32(14.4)	Ref		Ref	
Yes	31(68.9)	14(31.1)	2.68(1.29–5.59)	**0.008**	2.73(1.26–5.92)	**0.011**
Pre-XDR_FQ_-TB						
No	213(81.3)	49(18.7)	Ref			
Yes	27(84.4	5(15.6)	0.81(0.30–2.20)	0.672		

n = number of cases; OR = odds ratio; CI = confidence interval; IQR = interquartile range; HIV = human immunodeficiency virus; TB = tuberculosis; pre-XDR_SLID_-TB = resistance to one or more second-line injectable drugs; pre-XDR_FQ_-TB = resistance to one or more fluoroquinolones.

**Table 4:** Factors associated with death among patients with extensively drug-resistant tuberculosis (n = 142).

Factors	Death		Univariate analysis		Multivariate analysis	
	No n (%)	Yes n (%)	OR (95% CI)	*p*-value	OR (95% CI)	*p*-value
Age (years), median (IQR)	39.0(17)	43.5(17)	1.02(0.99–1.05)	0.309		
Gender						
Female	32(86.5)	5(13.5)	Ref			
Male	76(72.4)	29(27.6)	2.44(0.87–6.88)	0.091		
Country of origin						
Native	88(78.2)	26(22.8)	Ref			
Foreign-born	20(71.4)	8(28.6)	1.35(0.54–3.43)	0.523		
HIV status						
Negative	76(77.6)	22(22.4)	Ref			
Positive	32(72.7)	12(27.3)	1.30(0.57–2.93)	0. 534		
Alcohol abused						
No	68(81.9)	15(18.1)	Ref			
Yes	31(72.1)	12(27.9)	1.76(0.74–4.19)	0.205		
Injectable drug used						
No	75(83.3)	15(16.7)	Ref		Ref	
Yes	26(65.0)	14(35.0)	2.69(1.15–6.33)	**0.023**	2.19(0.80–5.96)	0.125
Imprisonment						
No	89(80.9)	21(19.1)	Ref		Ref	
Yes	9(56.2)	7(43.8)	3.30(1.10–9.87)	**0.033**	2.24(0.66–7.61)	0.198
Community residence						
No	94(79.7)	24(20.3)	Ref			
Yes	5(83.3)	1(16.7)	0.78(0.09–7.02)	0.827		
Homelessness						
No	97(80.8)	23(19.2)	Ref			
Yes	2(50.0)	2(50.0)	4.22(0.56–31.54)	0.161		
Comorbidities						
No	90(74.4)	31(25.6)	Ref			
Yes	18(85.7)	3(14.3)	0.48(0.13–1.76)	0.270		
Chest radiography						
No cavitation	48(82.8)	10(17.2)	Ref			
Cavitation	49(72.1)	19(27.9)	1.86(0.79–4.41)	0.158		
Previous TB treatment						
No	51(79.7)	13(20.3)	Ref			
Yes	57(73.1)	21(26.9)	1.45(0.66–3.18)	0.360		
Site of disease						
Pulmonary	99(75.6)	32(24.4)	Ref			
Extra-pulmonary	8(80.0)	2(20.0)	0.77(0.16–3.83)	0.753		

n = number of cases; OR = odds ratio; CI = confidence interval; IQR = interquartile range; HIV = human immunodeficiency virus; TB = tuberculosis.

Among MDR-TB patients, HIV infection (OR 4.14; 95% CI 2.21–7.74; *p* < 0.001), injectable drug use (OR 6.30; 95% CI 2.98–13.34; *p* < 0.001), presence of comorbidities (OR 2.31; 95% CI 1.11–4.81; *p* = 0.026) and resistance to one or more second-line injectable drugs (OR 2.68; 95% CI 1.29–5.59; *p* = 0.008) were significantly associated with death in univariate analysis. HIV infection (OR 4.55; 95% CI 2.31–8.99; *p* < 0.001) and resistance to one or more second-line injectable drugs (OR 2.73; 95% CI 1.26–5.92; *p* = 0.011) remained independently associated with death in multivariate analysis (Table 3).

Injectable drug use (OR 2.69; 95% CI 1.15–6.33; *p* = 0.023) and history past or present of imprisonment (OR 3.30; 95% CI 1.10–9.87; *p* = 0.033) were significantly associated with death among patients with XDR-TB in univariate analysis. However, these associations were not confirmed in the multivariate analysis (Table 4).

### The elapsed time between beginning of treatment and death

In the cases for which death was reported, the treatment duration until death was 9.5 months among MDR-TB patients and 13.1 months among XDR-TB patients (Table 5). In addition, 22 (40.7%) of the MDR-TB patients versus 8 (23.5%) of the XDR-TB patients died within the first six months (Table 5).

**Table 5:** Treatment duration until patient death among multidrug-resistant and extensively drug-resistant tuberculosis patients in cases where death was reported (n = 88).

Categories	Time in months, median (IQR)	Death	
		Within the first six months of treatment, n (%)	After the first six months of treatment, n (%)
MDR-TB	9.5(14.0)	22(40.7)	32(59.3)
XDR-TB	13.1(17.7)	8(23.5)	26(76.5)

IQR = interquartile range; n = number of cases; MDR-TB = multidrug-resistant tuberculosis; XDR-TB = and extensively drug-resistant tuberculosis.

We compared the demographic characteristics of MDR-TB patients who died within the first six months of treatment with patients who died after that period (S2 Table). Alcohol abuse (60.0%), injectable drug use (55.6%), and comorbidities (61.5%) were more frequent among MDR-TB patients that died within the first six months, although these differences were not statistically significant. The proportion of MDR-TB patients without additional second-line injectable drug resistance was significantly higher among them (56.2%; *p* = 0.020) (S2 Table).

## Discussion

This study evaluated treatment outcomes of a cohort of 436 patients with drug-resistant TB diagnosed in Portugal within 17 years, using national TB surveillance data. We paid special attention to death during treatment and identified factors associated with it in MDR- and XDR-TB patient groups.

We found that treatment was more successful for MDR-TB than XDR-TB patients. The case-fatality rate was highest among XDR-TB patients. Death during treatment occurred earlier for patients with MDR-TB than for the ones with XDR-TB; 40.7% of them died within the first six months of treatment. HIV infection and resistance to one or more second-line injectable drugs were independently associated with death among MDR-TB patients.

In our study, the overall treatment success rate among MDR/XDR-TB was 70.2%, below the 2020 target of the action plan for the WHO European Region (75%) [15]. However, treatment success rate among MDR-TB patients (77.9%) was higher than one the reported for the European Union by the European Centre for Disease Prevention and Control (ECDC) (48.1%) [2]. In this meta-analysis, 74 different studies were from several locations (60%) [16] and a study from Brazil (58,1%). Still, it was lower than reported by European countries, like Italy [17] and the Netherlands [18] (81.3% and 88%, respectively).

The treatment success rate among XDR-TB patients (62.8%) was higher than the one reported by the ECDC (37.4%) [2], in the meta-analysis mentioned above [16] and a study from Brazil [19] (26% and 18.6%, respectively).

*Death* was more frequent among unfavorable treatment outcomes in both patient groups. A higher frequency of *death* among MDR-TB patients was also reported by ECDC [2], while *lost to follow-up* was more frequently reported in the meta-analysis mentioned above [16], in Brazil [20] and China [21]. The case-fatality rate in our study was 18.4% that is equal to the rate reported in a meta-analysis (18%) [16], but is higher than reported by ECDC (17.1%) [2] and shown in Brazil (14.3%) [19] and China (2.8%) [21]. Nevertheless, in Pakistan, the rate was even higher (19.8%) [22].

Among XDR-TB patients, contrary to our results, *treatment failure* was a more frequent unfavorable outcome reported by ECDC [2] and shown in a meta-analysis [16] and Brazil [19]. However, our case-fatality rate (23.4%) is lower than was shown in Brazil (30.0%) [19], but is higher than was reported by ECDC (21.8%) [2] and in a meta-analysis (21%) [16].

We found that HIV infection was independently associated with death among MDR-TB patients. Although HIV infection was less prevalent among MDR-TB patients than among XDR-TB patients (23.8% *vs*. 31.0%), HIV infected patients of this group were 4.6 times more likely to die. This finding is consistent with the results described previously in several studies. A meta-analysis noted an increase in deaths among HIV-MDRTB co-infected patients in low-income regions compared with high-income regions [23]. In India and Tanzania, HIV infection was also associated with death [24,25]. Toxicity and adverse events from antiretroviral therapy (ART) coupled with MDR-TB, therapy’s side effects can be accountable for poor treatment outcome in this patient group. According to WHO, antiretroviral therapy must be started as early as possible (within the first eight weeks) after the beginning of anti-TB treatment, irrespective of CD4 T cell count [13]. However, a study carried out in South Africa found that ART use before MDR-TB treatment was significantly associated with higher case-fatality rate than when ART was initiated after the beginning of MDR-TB treatment [26]. Unfortunately, we do not assess information about antiretroviral therapy or CD4 cell counts because this information is not reported to our TB Surveillance System.

Resistance to one or more second-line injectable drugs was also independently associated with death among MDR-TB patients. We included this variable in the analysis of factors associated with death after observing the low treatment success rate (55.6%) and the high case-fatality rate (31.0%) among these patients that is contradictory to what has been demonstrated in previous studies [27,28].

The second-line injectable drugs (amikacin, kanamycin, and capreomycin) are some of the core second-line drugs used in the intensive phase of treatment of MDR-TB, which varied from 6 (WHO guidelines, 2008) [11] to 7–8.5 months (WHO guidelines, 2011) [12]. However, the role in treatment and the importance of resistance to each of these drugs is not the same. On the one hand, resistance to capreomycin [29] and kanamycin [9] were independently associated with unfavorable outcome in some studies. On the other hand, a recent meta-analysis showed that amikacin provided modest benefits in treatment, while kanamycin and capreomycin were associated with unfavorable outcomes [30]. Thus, according to the new WHO consolidated guidance of 2019, kanamycin and capreomycin are not included in longer treatment regimens [13].

Among XDR-TB patients, injectable drug use and being prisoner were associated with death in univariate analysis. However, all seven prisoners, who died, were also injectable drug users. Thus, a combination of these two factors should be considered as a risk situation for death in this group of patients.

Finally, we found that 40.7% of the MDR-TB patients, who died during treatment, died in the first six months. These patients represent more than half (56.2%) of the patients without additional second-line drug resistance who died during treatment. Alcohol abuse (60.0%), injectable drug use (55.6%), and comorbidities (61.5%) were also most frequent among them. These findings indicate that patients’ death is not due to resistance to second-line injectable drugs but due to their addictions and comorbidities.

One of the strengths of this study is that it evaluated treatment outcomes of a cohort of drug-resistant TB patients over a significant amount of time. These patients were also previously characterised for the genetics of the pathogen [31] and for their spatial distribution in Portugal [32]. The knowledge about risk factors for death generated in this study will improve patients’ clinical management, enhancing treatment success, which is another strength. One of the study’s limitations is a restriction of the studied variables due to retrospective study design and using only the SVIG-TB date. Thus, we could not assess the effect of important variables on lethality, such as antiretroviral therapy and CD4 T cell count.

In conclusion, our findings suggest that factors and conditions as HIV infection, injectable drug use, alcohol abuse and comorbidities are most often associated with death early on and during treatment. This suggests the need for early diagnosis of MDR-TB and further monitoring of patients that present those characteristics from treatment initiation. Furthermore, it is also required careful assessment of the relationship between early death and delayed MDR-TB diagnosis and clinical status of the patient that should grant further investigation.

## Supporting information

S1 Fig Treatment success rate by year, 2000–2016. (TIF)

S1 Table Treatment outcomes by drug resistance categories who started treatment between 2000 and 2016 (n = 436). n = number of cases; MDR-TB = multidrug-resistant tuberculosis; pre-XDR_SLID_-TB = pre-extensively second-line injectable drug-resistant tuberculosis; pre-XDR_FQ_-TB = pre-extensively fluoroquinolone-resistant tuberculosis; XDR-TB = and extensively drug-resistant tuberculosis. ^a^ Treatment success included only “Treatment completed” because no cured was registered. ^b^ Fisher’s Exact Test.
(DOCX)

S2 Table Characteristics of multidrug-resistant tuberculosis patients who died within and after the first six months of treatment (n = 54). ^a^ Not applicable for age. n = number of cases; IQR = interquartile range; HIV = human immunodeficiency virus; TB = tuberculosis.
(DOCX)

## Abbreviations

CI: confidence interval
DOT: Directly Observed Therapy
DST: drug susceptibility testing
ECDC: European Centre for Disease Prevention and Control
HIV: human immunodeficiency virus
INSA: Instituto Nacional de Saúde Ricardo Jorge
IQR: interquartile range
MDR-TB: multidrug-resistant tuberculosis
Mtb: *Mycobacterium tuberculosis*
OR: odds ratio
pre-XDR_FQ_-TB: pre-extensively drug-resistant tuberculosis resistant to either a fluoroquinolone
pre-XDR_SLID_-TB: pre-extensively drug-resistant tuberculosis resistant to second-line injectable drugs
TB: tuberculosis
WGS: whole-genome sequencing
WHO: World Health Organization
XDR-TB: extensively drug-resistant tuberculosis
