# Supplementary figures and images for: Evaluation of drug-resistant tuberculosis treatment outcome in Portugal, 2000–2016
Source: PLoS One. 2021 Apr 20;16(4):e0250028. doi: 10.1371/journal.pone.0250028 (PMC8057584; doi:10.1371/journal.pone.0250028)

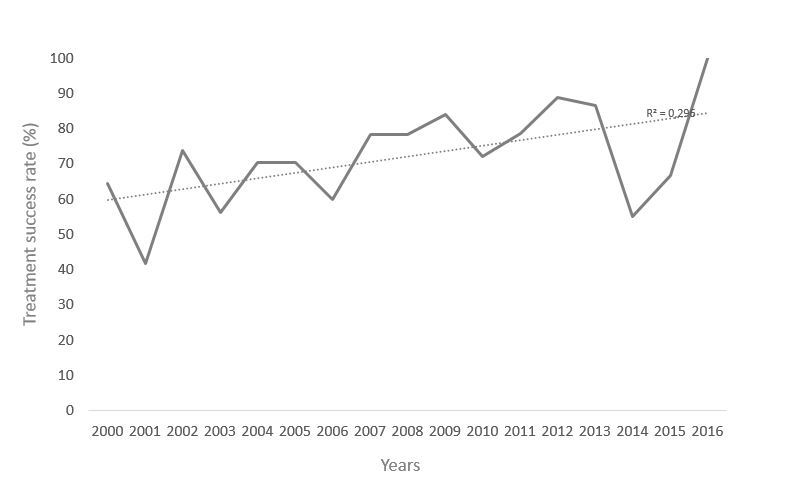

Supplement: S1 Fig — (TIF) [file pone.0250028.s001.tif]
