# Supplementary material for: Evaluation of drug-resistant tuberculosis treatment outcome in Portugal, 2000–2016
Source: PLoS One. 2021 Apr 20;16(4):e0250028. doi: 10.1371/journal.pone.0250028 (PMC8057584; doi:10.1371/journal.pone.0250028)
Supplement: S2 Table — a Not applicable for age. n = number of cases; IQR = interquartile range; HIV = human immunodeficiency virus; TB = tuberculosis. (DOCX) [file pone.0250028.s003.docx]

**S2 Table. Characteristics of multidrug-resistant tuberculosis patients who died within and after the first six months of treatment (n=54).**

| **Patient’s characteristics** | | **Death** | | | | ***p*-value** |
| --- | --- | --- | --- | --- | --- | --- |
|  |  | **Within the first six months of treatment** | | **After the first six months of treatment** | |  |
|  |  | n^a^ | IQR or % | n^a^ | IQR or % |  |
| Age (years) | Median, IQR | 44.0 | 27.0 | 39.5 | 30.0 | 0.481 |
| Gender | Female | 5 | 38.5 | 8 | 61.5 | 1.000 |
|  | Male | 17 | 41.5 | 24 | 58.5 |  |
| Country of origin | Native | 18 | 46.2 | 21 | 53.8 | 0.319 |
|  | Foreign-born | 4 | 26.7 | 11 | 73.3 |  |
| HIV status | Negative | 9 | 32.1 | 19 | 67.9 | 0.290 |
|  | Positive | 13 | 50.0 | 13 | 50.0 |  |
| Alcohol abuse | No | 12 | 32.4 | 25 | 67.6 | 0.150 |
|  | Yes | 6 | 60.0 | 4 | 40.0 |  |
| Injectable drug use | No | 8 | 28.6 | 20 | 71.4 | 0.128 |
|  | Yes | 10 | 55.6 | 8 | 44.4 |  |
| Imprisonment | No | 15 | 38.5 | 24 | 61.5 | 1.000 |
|  | Yes | 2 | 50.0 | 2 | 50.0 |  |
| Community residence | No  Yes | 18 | 45.0 | 22 | 55.0 | 1.498 |
|  |  | 0 | 0.0 | 2 | 100.0 |  |
| Homelessness | No  Yes | 17 | 41.5 | 24 | 58.5 | 0.429 |
|  |  | 1 | 100.0 | 0 | 0.0 |  |
| Comorbidities | No  Yes | 14 | 34.1 | 27 | 65.9 | 0.153 |
|  |  | 8 | 61.5 | 5 | 38.5 |  |
| Chest radiography | No cavitation  Cavitation | 13 | 52.0 | 12 | 48.0 | 0.099 |
|  |  | 5 | 23.8 | 16 | 762 |  |
| Previous TB treatment | No | 15 | 48.4 | 16 | 51.6 | 0.295 |
|  | Yes | 7 | 30.4 | 16 | 69.6 |  |
| Site of disease | Pulmonary | 20 | 40.0 | 30 | 60.0 | 1.000 |
|  | Extra-pulmonary | 2 | 50.0 | 2 | 50.0 |  |
| Pre-XDR_SLID_-TB | No | 18 | 56.2 | 14 | 43.8 | **0.020** |
|  | Yes | 2 | 14.3 | 12 | 85.7 |  |
| Pre-XDR_FQ_-TB | No | 21 | 42.9 | 28 | 57.1 | 0.638 |
|  | Yes | 1 | 20.0 | 4 | 80.0 |  |

^a^ Not applicable for age.

n= number of cases; IQR= interquartile range; HIV =human immunodeficiency virus; TB=tuberculosis.
